# Unusual microwave heating of water in reverse micellar solution

**DOI:** 10.1038/s41598-023-31742-1

**Published:** 2023-03-28

**Authors:** Hiroshi Murakami

**Affiliations:** Institute for Quantum Life Science, National Institutes for Quantum and Radiological Science and Technology (QST), Kyoto, 619-0215 Japan

**Keywords:** Biological techniques, Biotechnology, Cell biology, Materials science, Nanoscience and technology, Chemistry, Catalysis, Environmental chemistry, Green chemistry, Process chemistry, Chemical synthesis, Biological physics, Chemical physics, Condensed-matter physics

## Abstract

Microwaves (MWs) are widely used for heating food, accelerating chemical reactions, drying materials, therapies, and so on. Water molecules absorb MWs and produce heat because of their substantial electric dipole moments. Recently, increasing attention has been paid to accelerating various catalytic reactions in water-containing porous materials using MW irradiation. Here, a critical question is whether water in nanoscale pores generates heat in the same way as liquid water. Is it valid that MW-heating behaviors of nanoconfined water are estimated solely by a dielectric constant of liquid water? There are almost no studies regarding this question. Here, we address it using reverse micellar (RM) solutions. Reverse micelles are water-containing nanoscale cages formed by self-assembled surfactant molecules in oil. We measured real-time temperature changes of liquid samples within a waveguide under MW irradiation at 2.45 GHz and at MW intensities of ~ 3 to ~ 12 W/cm^2^. We found that the heat production and its rate per unit volume of water in the RM solution are about one order of magnitude larger than those of liquid water at all the MW intensities examined. This indicates that water spots that are much hotter than liquid water under MW irradiation at the same intensity, are formed in the RM solution. Our findings will give fundamental information to develop effective and energy-saving chemical reactions in nanoscale reactors with water under MW irradiation, and to study MW effects on various aqueous mediums with nanoconfined water. Furthermore, the RM solution will serve as a platform to study the impact of nanoconfined water on MW-assisted reactions.

## Introduction

Microwaves (MWs) are commonly used for modern life. They are electromagnetic waves in the frequency range of 0.3–300 GHz, corresponding to wavelengths of 1 mm to 1 m. MW ovens heat food effectively. Mobile phones use wireless communication relying on MWs. Moreover, applications of MW heating in chemical synthesis have been rapidly spreading, for example, to achieve green chemistry^1–6^.

MW heating of water takes place because of substantial interaction between MWs and water. MW ovens mainly use this mechanism because food contains water. Microwave-accelerated homogeneous catalysis in water has significant advantages because of high yields for a short time, compared with conventional heating^3–5^. Water molecules move with the time-dependent oscillatory electric field of applied MWs, producing heat owing to viscous friction between water molecules. Although such a rough description is provided, it is challenging to specify molecular processes involved in the heat production^7^. One reason for this difficulty is that systems under continuous MW irradiation are in non-equilibrium states^8^.

MW heating of aqueous mediums with nanoconfined water, which are made naturally or artificially, is widely used in many disciplines. For example, catalytic reactions in porous materials with nanoconfined water under MW irradiation were extensively studied toward fast, green and efficient synthesis and toward removal of organic pollutants in water ecosystems^9–12^. Moreover, MW synthesis of single-crystal porous transition-metal nitrides demonstrated that hydrated metal oxides are a new class of MW absorbing media, suggesting that nanoscale lattice water in the sample plays a crucial role in MW heating of the samples^13^. Another example is MW irradiation of organisms^14–20^ and fresh foods^21^ with the aims of drying, thawing, microbial inactivation, therapy, and so on, where intracellular water is ubiquitously confined on the nanoscale owing to the so-called molecular crowding^22^. Here, a fundamental question is raised on whether nanoconfined water generates heat in the same way as liquid (bulk) water. It, however, has never been addressed, and hence, the response of nanoconfined water to MWs is regarded as merely that of a very small amount of water with a dielectric constant of liquid water. We address this question here using reverse micellar (RM) solutions.

**Figure 1:**
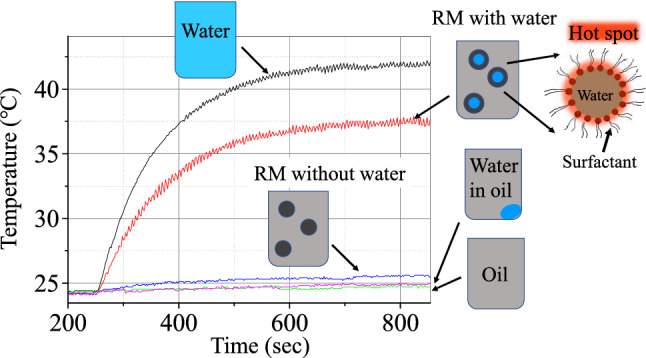
Real-time changes in the temperatures of liquid samples (volumes of 0.2 ml) under microwave (MW) irradiation (2.45 GHz, ~ 12 W/cm^2^). The samples were water (black), oil of isooctane (green), reverse micellar solutions with water (red) and without it (blue), and water in oil (red purple). A cross-section diagram of an RM with water is shown as a closeup on the right side.

A reverse micelle (also abbreviated as RM) is a nanoscale water droplet covered by a membrane of self-assembled surfactant molecules in a nonpolar (oil) solvent^23^, as displayed in Fig. 1. The RM size (nm–sub-µm) can be controlled by adjusting the water-to-surfactant molar ratio *w*_0_ (= [water]/[surfactant]), and it increases with* w*_0_. In addition, water-soluble molecules, such as dye and protein molecules, can be dissolved within RMs. Therefore, confinement effects on the structure^24–29^ and dynamics^30–36^ of water and on chemical and biochemical reactions^24,37–44^ of molecules encapsulated have been extensively studied for several decades, mostly by using RMs with a surfactant of AOT [= bis(2-ethylhexyl) sulfosuccinate] because of their stability over a wide size range.

## Results and discussion

Real-time changes in the temperatures of liquid samples measured by infrared thermography under MW irradiation are depicted in Fig. 1, where the experimental conditions were the same for all the samples. These results were confirmed by repeating the measurements at least a few times. The temperature (black line) of water substantially increases with time and reaches the stationary state at ~ 42 °C owing to being balanced against the heat dissipation to the external atmosphere. On the contrary, oil of isooctane does not show an increase in the temperature (green line), which is because isooctane is a non-polar molecule and has almost no interaction with the MW. Moreover, the temperature (blue line) of a sample of RM solution without water (*w*_0_ = 0) increases slightly by ~ 1 °C, where RMs are dispersed in oil, as schematically displayed in Fig. 1. A dramatic change in the temperature (red line) is obtained if a small amount of water [volume fraction (VF) of 0.025] is added to the RM solution without water, where the RM size grows from ~ 3 nm at *w*_0_ = 0 to ~ 5 nm at *w*_0_ = 7^25,26^. The temperature attains to ~ 37.5 °C, which is comparable with that of the sample of water. Here, the temperature recorded for the sample of RM solution with water is attributed to averaging the temperatures of water-containing RMs which absorb MWs and of the oil medium which is transparent to MWs. For comparison, the measurement was made for water (VF of 0.025) in isooctane (red purple line), where a water droplet was located at the bottom of the sample cell owing to phase separation with the oil. As described in Sect. 3 of SI, it was found that the temperatures hardly depend on the positions at which they are recorded, because of the small dimensions of the liquid sample. This also holds true for the sample of water in oil, in which the spatial distribution of water is not uniform. This sample contains the same amount of water as that in the RM solution with water, but displays almost no change in the temperature. This is understood by considering that the whole liquid is not heated by the small amount of water absorbing the MW. The result of RM solution with water is in marked contrast to this result.

The heat productions of samples of water and RM solution with water can be roughly estimated by specific heats of water and isooctane, 4.18 Jg^−1^ K^−1^ and 2.12 Jg^−1^ K^−1^, respectively, which do not so much vary in this small temperature range. In addition, the specific heat of isooctane is used for that of the RM solution containing isooctane with VF of ~ 0.9. The heat production of the sample of water is ~ 14.6 J for temperature change from 24.5 to 42 °C. On the other hand, it due to water in the RM solution is ~ 3.5 J from 25.5 to 37.5 °C, by using a density of 0.69 g/ml for isooctane, and by taking account of increase (~ 1 °C) in temperature due to the surfactant. The heat production of water in the RM solution is about one fourth of that of liquid water, whereas the amount of water in the RM solution is only ~ 2.5% of the sample of water. This indicates that the heat production per unit volume of water in the RM solution is about ten times larger than in liquid water. This highly effective MW heating of water in RM solution was further examined with changing the MW intensity, as described below.

**Figure 2:**
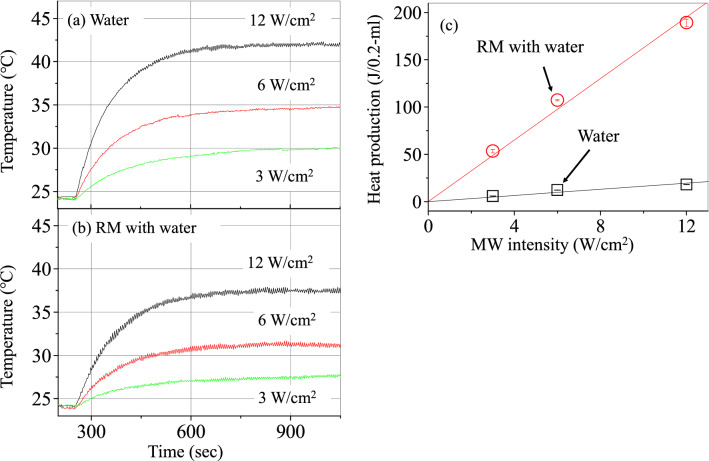
Real-time changes in the temperatures of samples of water (**a**) and RM solution with water (**b**) at three MW intensities, where black, red, and green curves are the results at MW intensities of 3, 6, and 12 (W/cm^2^), respectively. (**c**) Heat productions (J/0.2-ml water) due to liquid water (black squares) and to water in RM solution (red circles) as a function of MW intensity, where the mean values with standard deviations (error bars) are shown, obtained from the measurements made three times. The straight lines (red for RM with water and black for water) were gained by least-squares fitting to the data with coefficients of determination of 0.98 for RM solution and of 0.88 for water.

Real-time changes in the temperatures for samples of water and of RM solution with water at three MW intensities are depicted in Fig. 2a,b, respectively. These results were confirmed by repeating the measurements three times. It is found that the temperatures in the stationary states increase with raising the MW intensity for the two samples. Heat production due to water of 0.2 ml, which was the volume of the sample, is calculated at the three MW intensities in the same way as in the preceding paragraph, whereas it for the RM solution is obtained by dividing the heat production thus calculated by 0.025 (VF of water). Moreover, the temperatures in the stationary states were corrected for the difference between the temperatures of the liquid sample and by the thermographic camera (see SI), although this correction does not have much effect on the following conclusions. The results are displayed in Fig. 2c. It is found from the straight lines in Fig. 2c that the heat production of water in the RM solution is proportional to the MW intensity, and is much larger than that of liquid water at the same MW intensity. That is, the ratios are ~ 9 at 3 W/cm^2^, ~ 9 at 6 W/cm^2^ and ~ 10 at 12 W/cm^2^, and the ratio between the slopes of the two lines is ~ 10. Thus, the heat production per unit volume of water in the RM solution is roughly ten times larger than in liquid water at the same MW intensity.

**Figure 3:**
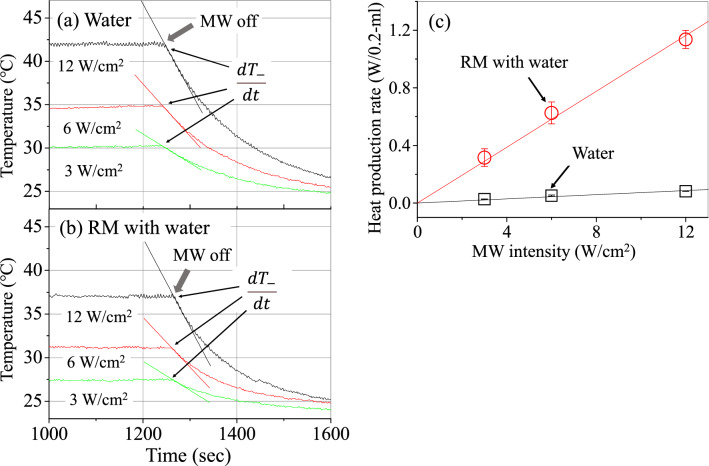
Real-time changes in the temperatures descending from the stationary states owing to turning off the MW for samples of water (**a**) and RM solution with water (**b**) at three MW intensities, where black, red, and green curves are the results at MW intensities of 3, 6, and 12 (W/cm^2^), respectively. The straight lines correspond to the first derivation (*dT*_/*dt*) at the time when the MW is turned off. (**c**) Heat production rates (W/0.2-ml water) due to water for liquid water (black squares) and for the RM solution (red circles) as a function of MW intensity, where the mean values with standard deviations (error bars) are shown, obtained from the measurements made three times. The straight lines (red for RM, black for water) were gained by least-squares fitting to the data with coefficients of determination of 0.97 for RM solution and of 0.92 for water.

The heat production rates are next estimated and compared between liquid water and water in the RM solution. The heat production rate is balanced against the heat dissipation rate in the stationary state, and hence the former is obtained as the value of the latter. The heat dissipation rate in the stationary state will be roughly estimated by the derivative value at the time when the MW is turned off. This is described in Fig. 3a,b for water and RM solution with water, respectively. These figures display the temperatures descending from the stationary states owing to turning off the MW irradiation. The slope of the straight line corresponds to the derivative value (${dT}_{-}/dt$). Moreover, by assuming that the time profile measured by the thermographic camera represents the real-time temperature change of the sample, the heat dissipation rate is obtained as the derivative value multiplied by the heat capacity of the liquid sample, where the heat capacity is calculated in the same way as in derivation of the heat production. The heat production rates (W/0.2-ml water) for water (squares) and for the RM solution (circles) are plotted as a function of the MW intensity in Fig. 3c. It is found from Fig. 3c that the heat production rate of water in the RM solution is proportional to the MW intensity, and is much larger than that of liquid water at the same MW intensity. That is, the ratios are ~ 12 at 3 W/cm^2^, ~ 12 at 6 W/cm^2^ and ~ 14 at 12 W/cm^2^, and the ratio between the slopes of the two lines is ~ 13. Thus, the heat production rate per unit volume of water in the RM solution is over ten times larger than that in liquid water at the same MW intensity.

There are many studies on water-in-oil (W/O) emulsions with MW irradiation^45–48^. These studies focus mainly on exploring effective separation of water from crude oil through the process of demulsification of W/O emulsions by MWs. However, the volume fractions (~ 0.2 to ~ 0.5) of water in those emulsions are much higher than that (~ 0.025) of the RM solution examined here, and the sizes of water droplets are in the micrometer range. Furthermore, there are no studies reporting unusual MW heating in those emulsions.

This unusual MW heating observed in RM solution with water is not explained by a dielectric loss of the sample. The heat production rate is usually regarded to be proportional to a dielectric loss, corresponding to the imaginary part ${\varepsilon }^{{\prime\prime}}$ of complex permittivity of a material^49^. The value of ${\varepsilon }^{{\prime\prime}}$ at 2.45 GHz and at room temperature is ~ 11 for water^50^, whereas it is ~ 0.05 for AOT RM solutions with a volume fraction of 0.1 and at *w*_0_ = 5 or 10^32^. These values of volume fraction and *w*_0_ are close to those (VF ~ 0.1, *w*_0_ = 7) of the RM solution examined in the present study, and hence, its ${\varepsilon }^{{\prime\prime}}$-value will also be close to that value. The very small permittivity of the RM solutions is regarded to be largely due to a low amount of water, and the ratio of ${\varepsilon }^{{\prime\prime}}$ value of the RM solution to liquid water is ~ 5 × 10^−3^. Thus, the ${\varepsilon }^{{\prime\prime}}$ value does not lead to an anomalous increase in the heat production rate obtained in the RM solution.

This unusual MW heating of the RM solution may be attributed to the following two considerations. One is an increase in the dielectric loss of the RM solution under MW irradiation. It is noteworthy that the permittivity is usually measured by a weak and perturbative MW irradiation, which allows us to observe materials in the thermal equilibrium and to obtain linear responses to external electric fields. On the contrary, a nonlinear response may emerge under strong MW irradiation that makes a system heated. For example, changes in the arrangement of water molecules may be induced, leading to a long-scale dynamic correlation over water molecules in the RM, which may help to sustain such a correlation owing to nanoconfinement. As a result, a growth of a dipole moment, which is ascribed to the correlation between the dipole moments of water molecules, may cause an increase in the dielectric loss of the RM solution. The other is Fresnel reflection of the MW at the interfaces^51,52^. The refractive indices of water, isooctane and quartz glass at 2.45 GHz are ~ 8.8, ~ 1.4, and ~ 1.9, respectively. The electromagnetic wave propagating from a medium with a large refractive index to that with a small one shows a total reflection at the interface. Therefore, the MW at 2.45 GHz will exhibit multiple reflections in water, reflected at the interfaces between water and glass for the sample of water, and between water and isooctane for the RM solution. The reflected MW will increase the total MW heating. Such enhancement may be a key factor for the unusual MW heating of RM solution with water. There are, however, theoretical challenges for clarifying it. The questions are, for example, how the MW is repeatedly reflected in the nanosized water, and how the water generates heat by the reflected MW. Multiphysics simulations of electromagnetic fields and heat transfer^53^ will be required to address these questions, and molecular dynamics simulations will need to be combined to examine it on the molecular level^8^.

It is considered that the RMs with water neither collapse nor form aggregates under MW irradiation, as described in SI. Therefore, nanoconfined water in the RM will be responsible for the dramatic increase in the temperature with MW irradiation, by taking account of the fact that this phenomenon is not observed for the RM without water.

Our findings show that water in RM solution exhibits significantly more effective MW heating than liquid water, and so becomes a hot spot. The temperatures of the RM solutions measured on the macroscopic scale under MW irradiation are moderate, at most ~ 38 °C. Thus, this study may provide a new aspect for understanding responses of various aqueous mediums with nanoconfined water, such as organisms and foods, to MW irradiation. For example, much attention has been paid to biological effects due to MW irradiation^14–20^. Non-thermal effects are often proposed for explaining phenomena such as changes in microorganisms’ activities^14–17^ and in cell membrane permeabilities^18^. Here, the temperatures of systems examined are controlled at given constant temperatures under MW irradiations. This experimental procedure is believed to validate the assumption that the MW effect is not due to heating. This occurrence, however, is not simple if there are hot spots, because the local excess heating may cause those biological effects. Furthermore, RM solution will allow us to study the impact of nanoconfined water on MW-assisted reactions because the chemical and biomolecular reactions can be examined in RMs^24,37–44^. Here, there are two advantages. One is in controlling their size, and the other is that oil solvents for RM solution are transparent to MWs.

## Methods

### MW heating experiment

**Figure 4:**
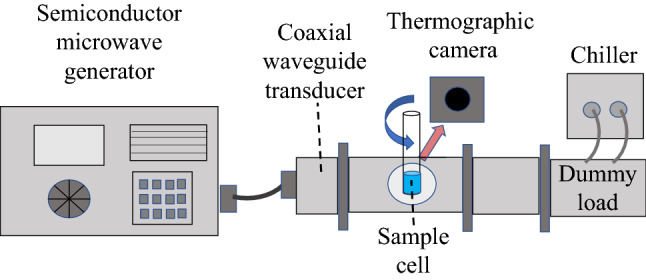
Schematic illustration of the measurement.

The experimental setup for MW heating is depicted in Fig. 4. A semiconductor microwave generator was used as a MW source at 2.45 GHz. The microwave was led to a waveguide with 27 × 96-mm^2^ cross-section from the MW generator through a coaxial waveguide transducer. This waveguide setup was constructed for a traveling wave mode, and a dummy load, which was coupled to a water-flow chiller, was put at the termination so that the MW passing through the sample can be dumped. The MW power dependence was measured at 75 W, 150 W, and 300 W, corresponding to 3 W/cm^2^, 6 W/cm^2^, and 12 W/cm^2^ on average, respectively; here, we note that most of the MW power applied was not used for heating liquid samples owing to their small dimensions.

**Figure 5:**
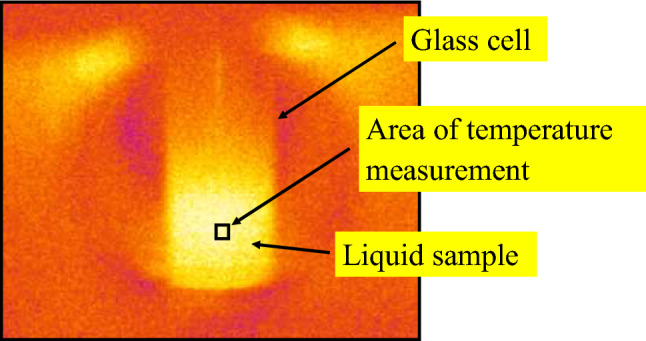
Thermographic image of a liquid-containing glass cell with MW heating.

A cylindrical quartz cell with 6-mm inner diameter was inserted into the waveguide through a hole in the top surface of the waveguide for heating liquid samples, and was set at the same position within the waveguide for all the samples by using a three-axes stage. Moreover, the sample cell was rotated at 700 rpm by a brushless motor, which was connected with the three-axes stage by a bracket, so that the sample can be uniformly heated. Nevertheless, almost no difference of the MW-heating behaviors was observed between the samples with and without the rotation of cell. This is because a penetration depth, which is defined as the distance from the surface of the material at which the power drops to ${e}^{-1}$ from its value at the surface, was larger than the inner diameter of the sample cell; as for liquid water, the penetration depth is ~ 15 mm^49^, and it is much larger for isooctane. The amounts of the liquid samples were 0.2 ml. An infrared thermographic camera (Optris, Germany) was used for temperature measurements of the liquid samples through a window in the side surface of the waveguide. The temperature was measured at the center of the liquid sample, as seen in Fig. 5, where the measured area was ~ 1 × 1 mm^2^. It was found that the temperatures hardly depend on the positions at which the temperatures are recorded, because of the small dimensions of the liquid samples (see Sec.3 in SI).

### Samples

Sample preparation of RM solution was described in detail elsewhere^29,36^. AOT [= bis(2-ethylhexyl) sulfosuccinate] (Sigma-Aldrich, USA), isooctane (Wako, Japan), and sterile Millipore-filtered water (Yamato, Japan) were used for a surfactant, oil and water, respectively. Isooctane is widely used to prepare stable AOT reverse micellar solutions over a wide RM size range. Moreover, its boiling point is ~ 99 °C, which is almost the same as that of water, at ambient conditions, and much higher than the temperature range examined. It is necessary to avoid boiling of RM solutions under MW heating.

The volume fractions of RMs with/without water were less than ~ 0.1 in the present study. Based on the previous studies^25,27,33^, this indicates that the RMs are homogeneously and separately distributed in the RM solutions, that is, each RM is thought to react to MWs.

A surfactant of AOT has an anionic hydrophilic head group, and its counter ion is a sodium cation. It is considered that the sodium cation does not contribute to MW heating of the RM solution examined, because the conductivity loss due to that cation is not observed for AOT reverse micellar solutions in the frequency range above ~ 0.01 GHz^32^.

## Supplementary Information

Supplementary Information.

## Data Availability

The data that support the findings of this study are available from the corresponding author upon reasonable request.
