# Supplementary material for: Unusual microwave heating of water in reverse micellar solution
Source: Sci Rep. 2023 Mar 28;13:5025. doi: 10.1038/s41598-023-31742-1 (PMC10050161; doi:10.1038/s41598-023-31742-1)
Supplement: Supplementary file 1 — Supplementary Information. [file 41598_2023_31742_MOESM1_ESM.docx]

**Supplementary Information**

**Unusual microwave heating of water in reverse micellar solution**

Hiroshi Murakami

Institute for Quantum Life Science, National Institutes for Quantum and Radiological Science and Technology (QST), Kyoto 619-0215, Japan

1. **Correction for temperatures measured by a thermographic camera**

To obtain information on the difference between the temperatures of the liquid samples and measured by the thermographic camera, the same sample cell as in the MW heating measurement, containing 0.2-ml water, was heated by a temperature-controlled hot plate. As the temperature of the hot plate was raised, the temperatures were measured by the thermographic camera and by a thermocouple dipped in water in the cell when the system reached the stationary state at each temperature. As shown in Fig.S1, the difference between the two temperatures becomes apparent at around 30 ℃, and grows gradually with increasing temperature. For derivation of the heat production in Fig.2(c), the temperatures of water in the stationary states were corrected by +1 ℃ at 3 W/cm^2^ and by +4 ℃ at 6 W/cm^2^ and 12 W/cm^2^, whereas those of RM solution with water were corrected


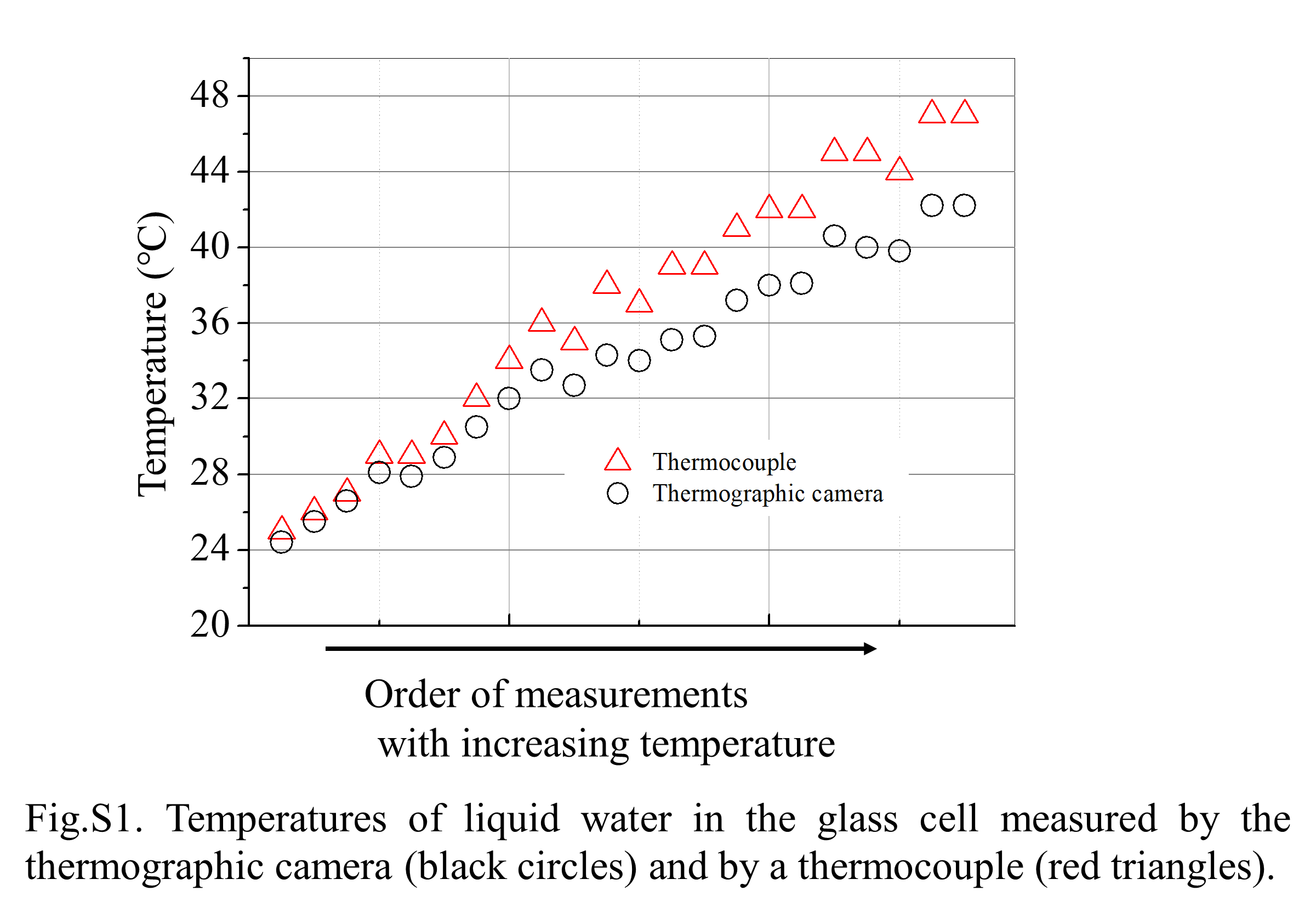
by +1 ℃ at 3 W/cm^2^, by +2 ℃ at 6 W/cm^2^ and by +4 ℃ at 12 W/cm^2^.

**2. Stability of RM solution with water**

We examined the stability of the sample of RM solution with water (*w*_0_ =7) at increasing temperatures or under MW irradiation, as follows.


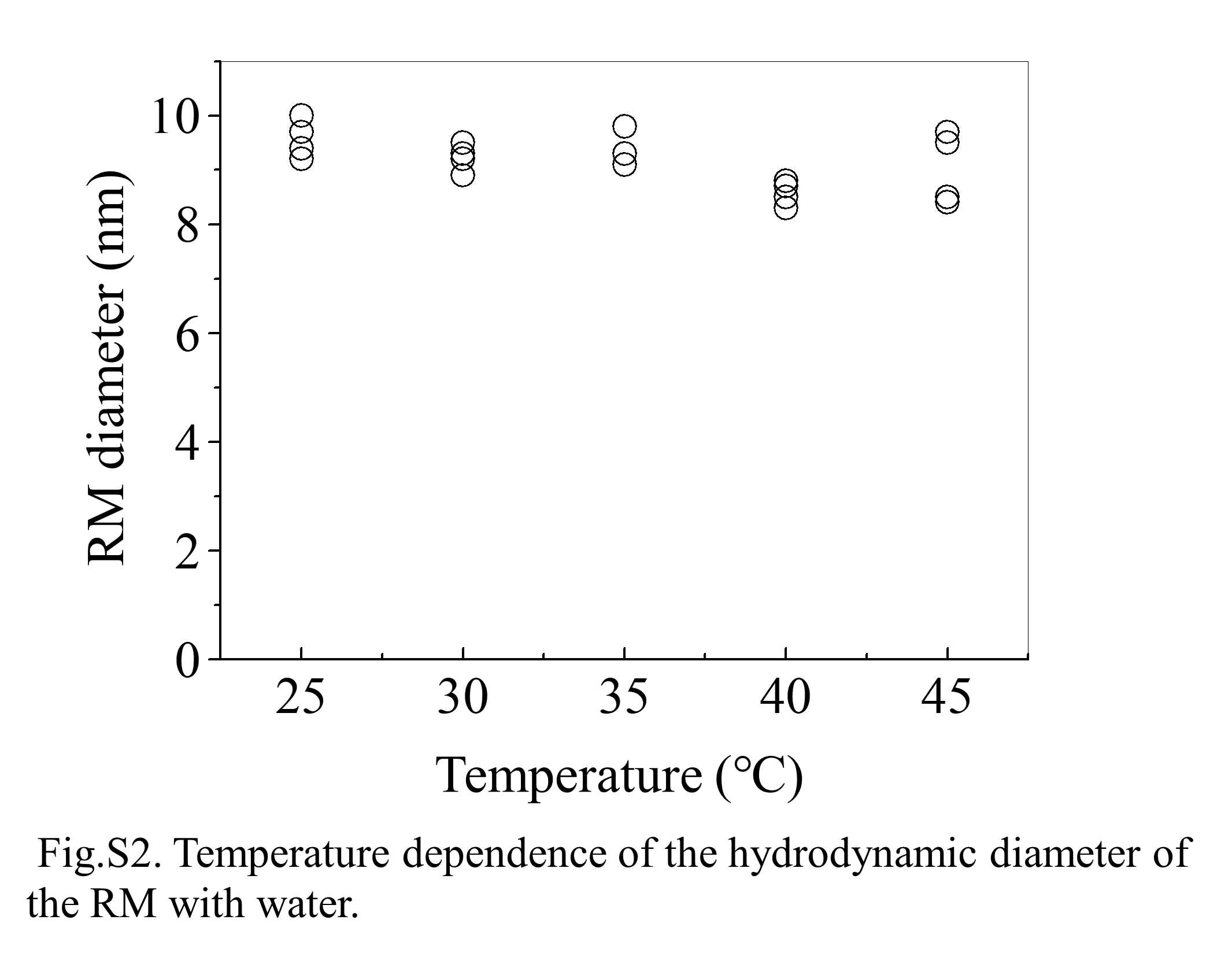
(1) Measurements using an equipment of dynamic light scattering (Photal, Japan) were made from 25 ℃ to 45 ℃ without MW irradiation, where the measurements were made several times at each temperature as the temperature was raised. Figure S2 shows that the hydrodynamic diameter of the RM with water hardly depends on temperature, and particles with larger sizes were not observed. This indicates that the RM is stable in the temperature range examined. If the RMs collapse, shedding water, and assemble into aggregates, the RM size decreases and larger-sized particles appear [36].

(2) The RM solution was clear just after the MW was turned off. The solution becomes opaque if RMs form aggregates [36]. The phase change to become opaque takes time (usually on a timescale of minutes), and hence, the RM solution will not be clear if the phase change takes place under MW irradiation; here we note that the measurements with MW irradiation were made on a timescale of minutes, which is long enough for exhibiting the phase change. Moreover, the temperature changes under MW irradiation were reproducible when we repeated the procedure in which the RM solution was heated by the MW and was cooled to room temperature by turning it off. This indicates that the RM solution is stable and does not exhibit irreversible changes under MW irradiation.

(3) The heat production rate on the way to the stationary state can be estimated roughly


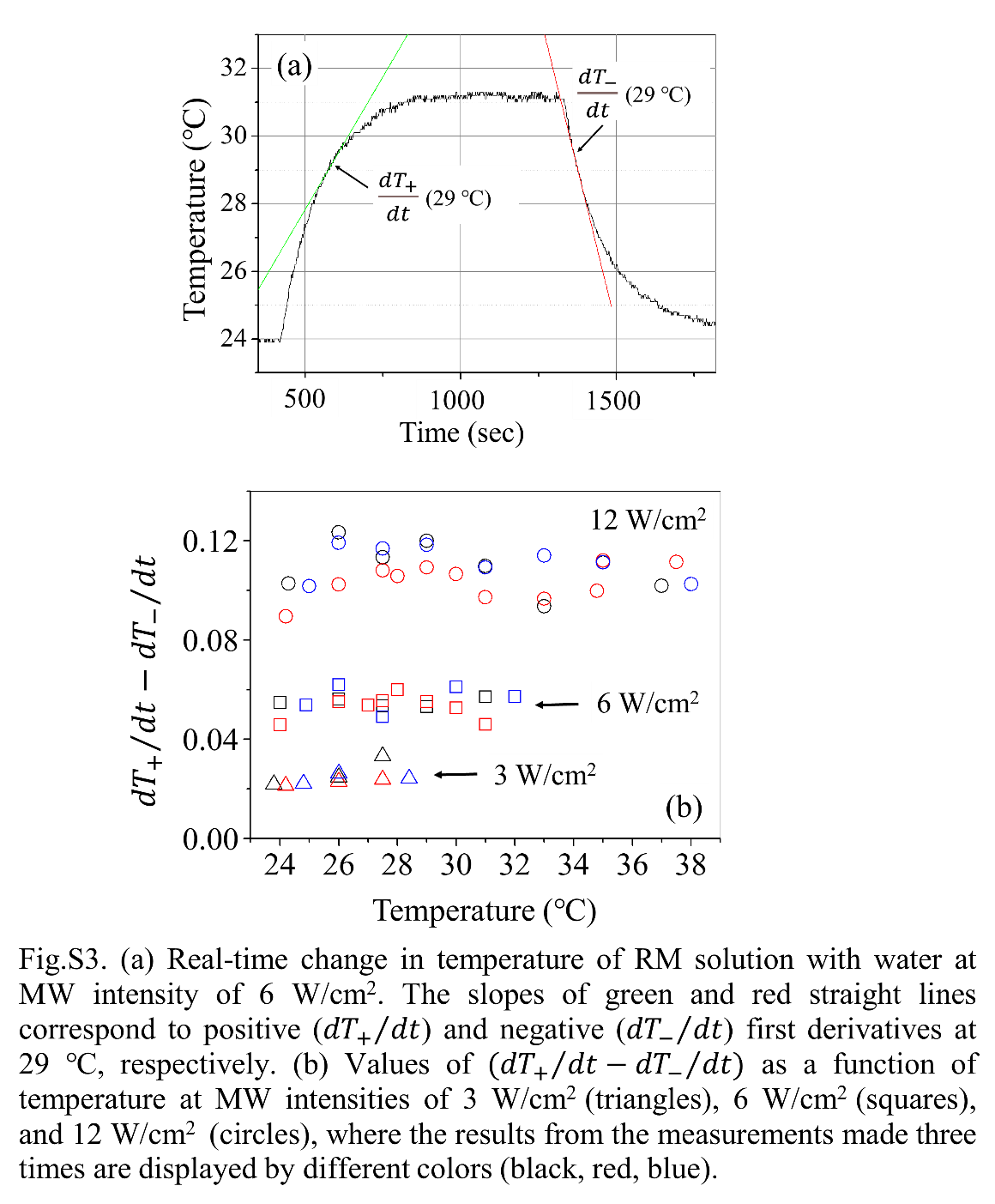
by ${C\times({dT}_{+}}/{dt}-{{dT}_{-}}/{dt)}$, where ${{dT}_{+}}/{dt}$and ${{dT}_{-}}/{dt}$ are positive and negative first derivatives, respectively, at the same temperature, as shown for the case at 29 ℃ in Fig.S3(a). $C$ is a heat capacity of the sample. Moreover, that rate at the start of MW heating can be estimated by ${C\times{dT}_{+}}/{dt}$ at the time when the MW is turned on. The temperature dependence of the heat production rate is represented by ${{dT}_{+}}/{dt}-{{dT}_{-}}/{dt}$, because the heat capacity hardly varies in this narrow temperature range. Values of ${{dT}_{+}}/{dt}-{{dT}_{-}}/{dt}$ at three MW intensities are plotted as a function of temperature in Fig.S3(b), where the results from the measurements made three times are superimposed. An increase in temperature in the abscissa represents an increase in time spent after the MW is turned on; for example, the temperature changed by ~1 ℃ in ~ 10 seconds after the MW was turned on at 12 W/cm^-1^. It is found from Fig.S3(b) that the values do not change from the start of MW irradiation to the stationary state at the maximum temperatures. This can be understood by considering that the water in the RM solution undergoes MW heating in the same way from the start of MW irradiation without changing the state of the RM.

There are many studies on water-in-oil (W/O) emulsions with MW irradiation, where the sizes of water droplets are in the micrometer range [45-48]. For example, it was demonstrated that the droplets of ~10 um collapse by MW irradiation, whereas they seldom do for ~1-um size even at 80 ℃, at a water volume fraction of 0.25, and without surfactants stabilizing emulsions [46]. In comparison with these previous studies, (1) the water-droplet sizes (several nanometers) are much smaller, (2) the temperatures (at most ~40 ℃) of RM solution with water are much lower, (3) the water volume fraction (0.025) is much lower, and (4) a surfactant is contained, in the present study. Thus, the previous studies of W/O emulsions seem to support our conclusion that the RMs do not collapse but are stable under MW irradiation, although the chemicals used are different from those in the present study.

**
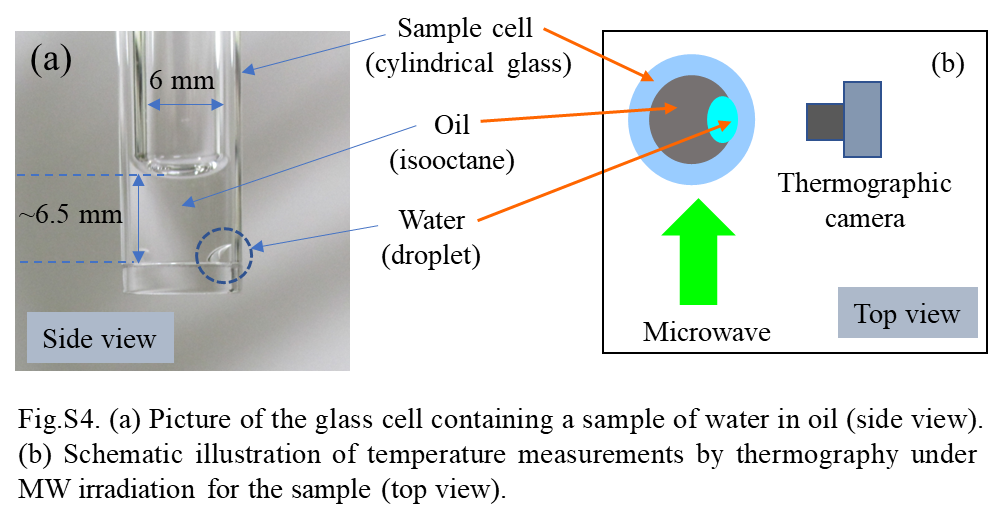
3.** **Position distributions of temperatures measured by thermography under MW irradiation**

In this section, we describe position distributions of temperatures measured by the thermographic camera in a vertical direction for samples of water in oil (isooctane) and RM solution with water (*w*_0_=7). In a sample of water in oil, the water droplet producing
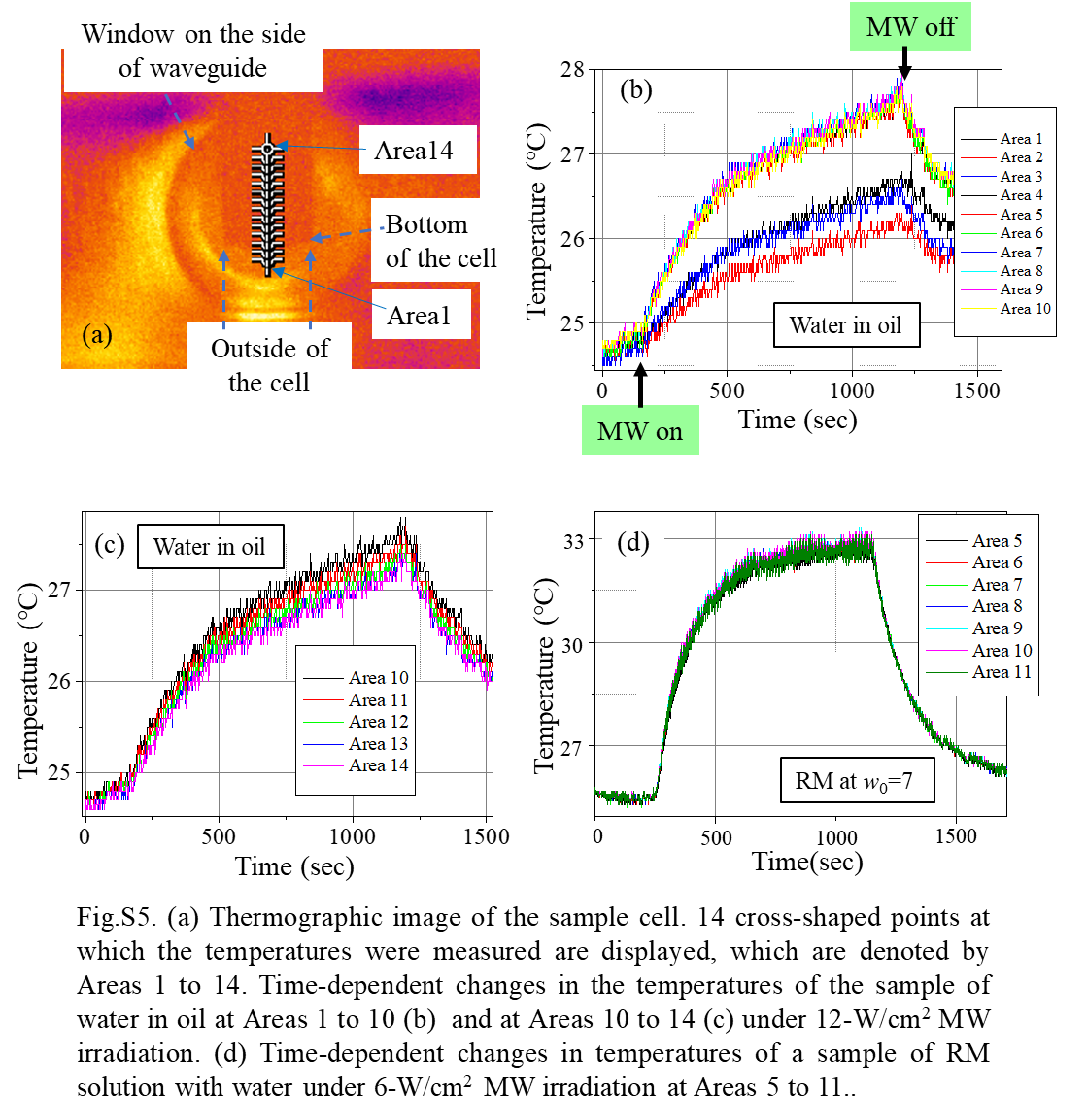
heat by absorbing MWs is located at the bottom of the sample cell and at its inner edge, as seen in Fig.S4(a). The configuration of temperature measurements by the thermography for the liquid sample is schematically depicted in Fig.S4(b). In this measurement, the sample cell was not rotated, the water droplet faced the camera, and MW was irradiated from the side. Figure S5(a) displays a thermographic image of the sample cell containing the liquid sample. The temperatures were measured at 14 points in a vertical direction, which are shown by cross-shaped points (denoted by Area No.). The distance between the adjacent points was ~0.9 mm, and the measurement range covering the height (~6.5 mm) of the liquid sample was ~12 mm. Time-dependent changes in the temperatures under MW irradiation of 12 W/cm^2^ at Areas 1 to 10 are depicted in Fig.S5(b). This figure indicates that the time behaviors at Areas 4 to 10 agree, whereas they disagree with those at Areas 1 to 3. The distance between Areas 4 to 10 is ~6.5 mm if the measured area (~1×1 mm^2^) at each point is taken into account, and corresponded to the height of the liquid sample. Therefore, it is considered that the temporal behaviors of the temperatures are almost independent of the positions at which they are recorded, as long as those positions are within the sample’s height. This will be because the thermal conduction from the water droplet to the whole oil is fast enough compared with the time resolution (2 sec) of the temperature measurement owing to the small dimension of the sample. On the other hand, it is reasonable to consider that Areas 1 to 3 are apart from the sample cell, and hence, these temperatures are lower than those at Areas 4-10. In Fig.S5(c), time-dependent changes of the temperatures measured at Areas 10 to 14 are displayed. It is found that the temperatures decrease gradually as the number of Area is increased, that is, as the position for the measurement is set away from the liquid, although the temperatures at Areas 10 and 11 are very close to each other. Here, we note that the environment of the experimental system exhibited a gradual increase in temperature because of heat from the MW generator. As seen in Figs.S5(b) and (c), the temperatures are affected by changes in the environmental temperature and do not reach the stationary states.

Figure S5(d) shows time-dependent changes in the temperatures of RM solution with water under MW irradiation of 6 W/cm^2^ at Areas 5 to 11, where the sample cell was rotated at 700 rpm. It is found that the temporal behaviors exhibit almost no dependence on the positions at which the temperatures are measured in the height of the liquid sample.
